# Disease characteristics of HLA-B27 positive and negative finnish patients with juvenile idiopathic arthritis - results of the 18-year cohort follow-up study

**DOI:** 10.1186/s12969-023-00878-9

**Published:** 2023-09-01

**Authors:** Suvi Oili Orvokki Peltoniemi, Mia Glerup, Pekka Lahdenne, Kari K Eklund, Kristiina Aalto

**Affiliations:** 1 Department of Rheumatology, Helsinki University Hospital and University of Helsinki, Helsinki, Finland; 2 Department of Paediatric and Adolescent Medicine, Aarhus University Hospital, Aarhus, Denmark; 3 Department of Paediatrics, New Children’s Hospital, Helsinki University Hospital and University of Helsinki, Aarhus, Finland

**Keywords:** Juvenile idiopathic arthritis, HLA-B27, Follow-up study, Remission

## Abstract

**Background:**

The aim of this long-term follow-up study was to compare the disease characteristics of HLA-B27 positive and negative patients with juvenile idiopathic arthritis (JIA).

**Methods:**

The study is a cohort study with consecutive cases of newly diagnosed Finnish patients with JIA according to the International League of Associations for Rheumatology (ILAR) criteria [1]. Patients were enrolled between 1997 and 2000 from a defined area of Southern Finland. Clinical data including disease activity and serology were registered during a mean period of 17.5 years.

**Results:**

159 patients completed the 18-year follow-up study. HLA-B27 was available for 151 patients, of which 25% were HLA-B27 positive. Chronic uveitis was diagnosed in 30% of HLA-B27 positive and 29% of HLA-B27 negative patients. HLA-B27 positive patients had a lower prevalence of temporomandibular (TMJ) involvement than the antigen negative ones, 19% versus 28%. None of the HLA-B27 positive patients had cervical spine affected compared to 11% of antigen negative patients (p = 0.022). Of the HLA-B27 positive patients, 54% had had biological medication at some point during follow-up versus 25% in the negative group (p = 0.003). At last follow-up, 32% of antigen positive patients were not in remission compared to 18% of the antigen negative (p = 0.017).

**Conclusions:**

The use of biological medication was more common in HLA-B27 positive patients with JIA. At the 18-year follow-up, more antigen positive patients had active disease compared HLA-B27 negative patients. This real-world follow-up study indicates that the prospects for worse outcome with HLA-B27 positivity in long-term should be taken into consideration.

## Background

Juvenile idiopathic arthritis (JIA) is an important cause of disability in children [2]. Long-term studies have shown that many patients still have persistent inflammation and disability in adolescents [3]. In the long-term, the disease may cause permanent joint damage, lower quality of life, and increased psychological burden [4, 5].

Because of the heterogeneity of JIA, there are not many clinical or laboratory parameters that could be used to predict the prognosis and severity of a patient’s disease. The human leukocyte antigen HLA-B27 is more frequent in patients with JIA than in the general population as described in an Estonian prospective JIA study where the proportion of patients with HLA-B27 was 28.6% [6]. At present HLA-B27 plays a primary role in the classification of JIA, being one of the inclusion criteria of enthesitis-related arthritis (ERA) category. In Nordic patients with JIA the presence of HLA-B27 has been shown to predict a more extended disease course with older age at disease onset in boys [7, 8]. However, the impact of HLA-B27 on the prognosis, disease characteristics and use of antirheumatic medication in JIA patients in the long-term is still incompletely known.

The aim of this study was to explore whether there are differencies in disease characteristics or prognosis between HLA-B27 positive and negative Finnish children with JIA during a long-term follow-up.

## Methods

### Patients

The patients were enrolled in a consecutive order based on a new diagnosis of JIA according to ILAR criteria [1]. The enrolment took place in Helsinki University Hospital (HUS) district area in Southern Finland between January 1997 and December 2000. All the patients were originally recruited to a Nordic follow-up study, but due to its later tightened enrolment period, some were left out and they continued only in a Finnish follow-up, but still based on the Nordic protocol. 61% of the patients participated also in the Nordic population-based 8-year JIA study [2] and 79% in the Nordic 18-year follow-up [9].

The 8-year follow-up visits took place in 2005–2008 and 18-year visits in 2014–2017. An option of standardized telephone interview was offered, if a participant was unable to attend a clinical study visit. In both cases, a thorough inquiry in reference to symptoms, medication, medical family history, and socioeconomic situation was performed. In clinical visits, data about joint examination and blood samples was collected. In addition, information regarding growth, visits at ophthalmologist and orthodontist, and intra-articular injections were registered. The MRI of SI joints was performed, if patients experienced lower back pain and there was a clinical suspicion of sacroiliitis. The evaluation of TMJ involvement was based on clinical examination and magnetic resonance imagining (MRI) or cone beam computed tomography (CBCT). The involvement of cervical spine was assessed by MRI after clinical examination in symptomatic patients. In addition, patient reported outcome measures were used.

### Laboratory tests

Routine laboratory parameters such as hemoglobin, C-reactive protein (CRP), erythrocyte sedimentation rate (ESR), and thrombocytes were followed. Furthermore, rheumatoid factor (RF), antinuclear antibodies (ANA), and HLA-B27 antigen were analyzed in most of the patients during the initial study period. ANA or RF positivity were defined in accordance with the ILAR classification [1]. Thus, two positive analyses were required with at least 3 months in between. The HLA-B27 was determined by PCR and agarose gel electrophoresis.

### Remission criteria

Patients were classified into various states of disease activity according to the criteria proposed by Wallace et al. [10]. Patients may have either active or inactive disease. The criteria for the latter include the following: no active arthritis; no fever, no rash, serositis, splenomegaly, or generalized lymphadenopathy attributable to JIA; no active uveitis; normal ESR or CRP level; and a physician’s global assessment of disease activity indicating clinical disease quiescence. Inactive disease can be divided further into clinical remission on medication (a minimum of 6 continuous months of inactive disease while receiving medication) and clinical remission off medication (12 months of inactive disease while not receiving any anti-arthritis or anti-uveitis medication).

### Statistical methods

Statistics program IBM SPSS Statistics 27 was used to analyse the collected data. Differences between two groups were tested with the chi-square or Fisher’s exact test as appropriate. When comparing non-parametric data between two different groups, a Mann-Whitney test was used. To assess correlation relationships between variables, a regression analysis was performed. When analyzing the variance between sex and the presence of HLA-B27, two-way ANOVA was used. P-values less than 0.05 were considered statistically significant.

## Results

187 JIA patients were originally recruited into the study. Of them 168 (89%) were followed for 8 years and 159 (85%) for a mean of 17.5 years. Of these 159 patients, HLA-B27 was available in 151 participants of whom 25% were HLA-B27 positive (19 females and 18 males) and 75% negative (74 females and 40 males). 33 patients (22%) were telephone interviewed.

Among the HLA-B27 positive patients after 8 or 18 years of disease onset, the most common ILAR category was ERA (37% or43%, respectively). Oligoarthritis (52% and 61%) was the most common category among the HLA-B27 negative patients, when combining the persistent and extended categories together (Table 1). There were no HLA-B27 negative patients with ERA. 3 (19%) of the 16 patients with ERA were females. Between 8 and 18 years the number of patients with juvenile psoriatic arthritis (jPsA) increased from 8 to 13 of whom 7 (54%) were female. 2 (15%) of the jPsA patients were HLA-B27 positive. 85% of jPsA patients had psoriasis and 36% of them had developed it later than 8 years after the disease onset.

**Table 1:** ILAR classification of 151 Finnish patients with JIA at 18-year follow-up

ILAR subgroup	HLA-B27 positive (% of the whole subgroup)	HLA-B27 negative (% of the whole subgroup)	n (% of total cohort)
Systemic disease	1 (3)	0 (0)	1 (1)
Oligoarticular persistent	3 (8)	41 (36)	44 (29)
Oligoarticular extended	3 (8)	28 (25)	31 (21)
Polyarticular RF neg.	9 (24)	30 (26)	39 (26)
Polyarticular RF pos.	1 (3)	2 (2)	3 (2)
Psoriatic arthritis	2 (5)	11 (10)	13 (9)
Enthesitis-related arthritis	16 (43)	0 (0)	16 (11)
Undifferentiated arthritis	2 (5)	2 (2)	4 (3)
Total	37	114	151

The involvement of TMJ was registered in 19% or 28% of the HLA-B27 antigen positive or negative patients, respectively. None of the antigen positive patients had involvement of the cervical spine, whereas 13 (11%) of the HLA-B27 negative patients had had cervical spine involvement (p = 0.022).

In the HLA-B27 positive or negative group, 73% or 89% of the patients had had arthritis of large joints during the 18-year follow-up period, respectively (p = 0.017). Small joint arthritis had been observed in 92% or 81% of the patients with or without HLA-B27, respectively (p = 0.086). The proportion of those with arthritis in the lower extremities was 95% in the antigen positive and 96% in the antigen negative group. Among the female patients, involvement of small joints in lower extremities correlated with HLA-B27 positivity (p = 0.021). In patients with or without HLA-B27, the percentage for having had arthritis in upper extremities was 57% or 61%, respectively. Hip joint involvement was observed in 19% or 21% of the HLA-B27 positive or negative patients, respectively. There was no statistically significant difference between the total cumulative number of joints affected between the positive and negative groups (Table 2).

**Table 2:** HLA-B27, sex distribution, and other characteristics of 151 Finnish patients with JIA during 18 years after disease onset

	HLA-B27 positive			HLA-B27 negative			Total		
	Female	Male	Total	Female	Male	Total	Female	Male	Total
n (%)	19 (51)	18 (49)	37	74 (65)	40 (35)	114	93 (62)	58 (38)	151
Median (mean) age at onset	6.2 (7.0)	10.1 (8.9)	9.0 (7.9)	3.0 (5.6)	5.2 (5.9)	5.0 (5.7)	4.7 (5.9)	6.4 (6.8)	5.2 (6.3)
Median number of cumulative joints (IQR)	6 (5)	4 (4)	5 (5)	7 (8)	6 (9)	6 (9)	6 (8)	5 (9)	6 (8)
TMJ involvement	5	2	7 (19%)	22	10	32 (28%)	27	12	39 (26%)
Cervical spine affected	0	0	0 (0%)	7	6	13 (11%)	7	6	13 (9%)
Hip joint affected	1	6	7 (19%)	17	7	24 (21%)	18	13	31 (21%)
Sacroiliitis on imagining	2	5	7 (19%)	1	2	3 (3%)	3	7	10 (7%)
Enthesitis 8–18 years	3	7	10 (27%)	1	1	2 (2%)	4	8	12 (8%)
Dactylitis	1	1	2 (5%)	7	3	10 (9%)	8	4	12 (8%)
Acute uveitis	1	3	4 (11%)	0	0	0 (0%)	1	3	4 (3%)
Chronic uveitis	6	5	11 (30%)	23	10	33 (29%)	29	15	44 (29%)

By 18 years, 10 of the 151 patients had had sacroiliitis on imaging (MRI or both MRI and x-ray) versus five by 8 years. Seven (70%) of them were HLA-B27 positive (p = 0.002). 3 of the 10 patients were females. 2 of the antigen negative patients with sacroiliitis had jPsA and 1 seronegative polyarthritis. None of the patients with sacroiliitis had had biological medication prior to its diagnosis. 38% or 31% of antigen positive or negative patients had had a tenosynovitis during the 8-year follow-up period, respectively (p = 0.271). Between 8 and 18 years, 27% of HLA-B27 positive patients had had enthesitis versus 2% of negative ones. By 18 years, 3 HLA-B27 positive males with ERA had been diagnosed to have inflammatory bowel disease (IBD).

During the 18-year follow-up, chronic uveitis was diagnosed in 30% or 29% of HLA-B27 positive or negative patients, respectively. ANAs were present in 55% of antigen positive uveitis patients versus 30% in the antigen negative ones (p = 0.102). None of the HLA-B27 negative patients had acute symptomatic uveitis compared to four of the HLA-B27 positive patients, who were all ANA negative. Having both positive ANAs and HLA-B27 did not show statistically significant correlation with the risk of uveitis (p = 0.541).

The most commonly used conventional disease modifying antirheumatic drugs (DMARDs) were methotrexate (MTX) and hydroxychloroquine (HCQ) (Table 3). At 8-year follow-up, sulfasalazine (SSZ) had been used by 21% of antigen positive versus 3% of antigen negative patients (p = 0.002). By 18 years, 27% of HLA-B27 positive versus 11% of negative patients had used SSZ (p = 0.025). At 8-year follow-up visit, 26% of HLA-B27 positive and 19% of HLA-B27 negative patients were using biological drugs (p = 0.556). At 18 years, 35% of the antigen positive and 14% of antigen negative patients had ongoing biologic treatment (p = 0.011). The biological medications used were mainly tumor necrosis factor alpha inhibitors (TNFi). At 18-year follow-up, only two patients were on other biologics: one patient was using rituximab and another abatacept. The majority (56%) of the HLA-B27 negative patients with ongoing biologics had seronegative polyarthritis and 46% of the antigen positive users of current biological medication had ERA. 54% of HLA-B27 positive versus 27% of negative patients had had biological medication at some point during the follow-up period (p = 0.003).

**Table 3:** Number of past and current users of traditional antirheumatics, steroids and biological medication at 18 years

Medicine	Used in the past		Used currently		Total number of users (%)
	HLA-B27+ (%)	HLA-B27- (%)	HLA-B27+ (%)	HLA-B27- (%)	
Steroids p.o.	22 (59)	48 (42)	1 (3)	3 (3)	74 (49)
Hydroxychloroquine	10 (27)	47 (41)	3 (8)	8 (7)	68 (45)
Sulfasalazine	8 (22)	5 (4)	2 (5)	8 (7)	23 (15)
Methotrexate p.o.	16 (43)	58 (51)	11 (30)	9 (8)	94 (62)
Methotrexate s.c.	5 (14)	17 (15)	1 (3)	5 (4)	28 (19)
Leflunomide	5 (14)	13 (11)	0 (0)	3 (3)	21 (14)
Other DMARDs*	3 (8)	5 (4)	1 (3)	1(1)	10 (7)
Biological medication	7 (19)	15 (13)	13 (35)	16 (14)	51 (34)
2 different biologics used	7 (19)	8 (7)			15 (10)
3 different biologics used	1 (3)	5 (4)			6 (4)
4 different biologics used	0 (0)	3 (3)			3 (2)

*Other DMARDs includes azathioprine, gold, mycophenolate, and cyclosporine.

** Used biological medications: Infliximab, etanercept, adalimumab, golimumab, rituximab, and abatacept.

At the 8-year follow-up, 39% of antigen positive patients and 32% of antigen negative ones were not in remission versus at 18-year follow-up visit, 32% in the HLA-B27 positive and 18% in the negative group (p = 0.017). Out of the antigen positive patients not in remission, 58% were females. At 18-year visit, 38% of ERA and 31% of jPsA patients had active disease. At 18-year follow-up visit of the HLAB-B27 positive or negative patients, 8 (22%) or 55 (48%), respectively, were in remission off medication (Table 4).

**Table 4:** Remission status of 151 Finnish patients with JIA at 18-year follow-up

	HLA-B27 positive (% of the whole subgroup)	HLA-B27 negative (%)	Total (%)
No remission*	12 (32)	21 (18)	33 (22)
Remission on medication	16 (43)	31 (21)	47 (31)
Remission off medication	8 (22)	55 (36)	63 (42)
Information missing	1 (3)	7 (5)	8 (5)
Total	37	114	151

*Either active joint disease or uveitis or both

At 18-year visit, 47% of patients in remission on medication were using biologics as mono- or combination therapy with traditional DMARDs. In the no remission group, 30% of the patients had used biologics in the past (p < 0.001). One third of antigen negative patients had had all their medication discontinued. However, 42% of antigen positive patients with active disease still had ongoing biological medication versus 14% of antigen negative.

During the whole follow-up period, seven patients had developed wrist erosions with rigidity, five of them were antigen negative. Two HLA-B27 positive patients had hip damage. Eight patients had micrognatia of whom six were HLA-B27 negative. There was no cervical ankylosis or atlanto-axial subluxation. Only one antigen negative patient had developed permanent limitation of cervical spine movement.

## Discussion

In this prospective long-term real-world study, some differences in the profile of affected joints between HLA-B27 positive and negative JIA patients were found. Cervical spine and large joints were affected less often in the antigen positive patients. In addition, ANA positive chronic uveitis seemed to be more common among HLA-B27 positive JIA patients.

Interestingly, the inflammation the cervical spine was more common in HLA-B27 negative patients. Majority of the studies regarding the cervical spine in JIA have been conducted before the availability of MRI, but already in 1988 Espada et al. noted the rarity of HLA-B27 positivity among juvenile rheumatoid arthritis patients with cervical spine involvement [11]. In this study, no differences were observed in the involvement of joints in upper or lower extremities or involvement of small joints in general, but larger joints were affected more often HLA-B27 negative patients. It is known that in addition to ERA patients, hip involvement is often seen in polyarticular disease and sometimes in sJIA [12, 13]. In a study published in 1991, shoulder joint was affected in majority of polyarticular and systemic onset juvenile rheumatoid arthritis patients [14].

Patients who had radiologically verified sacroiliitis 8 years after disease onset were all HLA-B27 positive boys with diagnosis of ERA. By 18 years, the number of patients with sacroiliitis had doubled including two antigen positive females with ERA and two antigen negative jPsA patients. Thus, axial involvement seems to be mostly associated with the HLA-B27 antigen, especially in lumbosacral region, but approximately half of the cases being diagnosed between 8 and 18 years after disease onset. The first study demonstrating the association between HLA-B27 and the late development of ankylosing spondylitis in children with arthritis was published already in the early 1970s [15]. Yet 50 years later, we still lack means to identify these patients developing axial disease early enough. Such patients would benefit from early intervention to prevent the disease progression into ankylosing spondylitis. None of the patients who developed sacroiliitis had used biological medication prior to that. Thus, it is possible that without biologics, there would have been even more cases of sacroiliitis.

Enthesitis was not one of the variables collected in the first 8 years of the study. Between 8 and 18 years, only 12 patients were registered to have had enthesitis. 9 of them had ERA and two patients jPsA. Enthesitis had a strong correlation with HLA-B27 positivity. Acute anterior uveitis and development of IBD were seen solely with HLA-B27 positive patients. Acute anterior uveitis is known to be the most common extra-articular manifestation of SpA in adults and it is also more frequent in HLA-B27 positive individuals even in the absence of arthritis or other features of SpA [16]. Furthermore, there is a strong link between gut inflammation and SpA: Subclinical gut inflammation is present in up to two thirds of all SpA patients and it can evolve into IBD [17].

Kotaniemi et al. reported the incidence of uveitis in Finnish patients with recently diagnosed juvenile chronic arthritis to be as high as 24% [18]. In the present study, the observed incidence of uveitis 18 years after disease onset was even higher (32%). Particularly, more cases of acute uveitis had appeared later between 8 and 18 years after onset. It has been shown earlier that JIA patients with acute symptomatic uveitis have their first attack of uveitis later in life than those with asymptomatic uveitis [19]. 31% of patients with uveitis were HLA-B27 positive, which is in line with previous Finnish studies [18, 19]. The overall frequency of HLA-B27 in the Finnish population is approximately 15% that is higher than in most of the other European populations [20, 21].

During the 18 years, MTX, SSZ and steroids were prescribed more often to antigen positive patients. However, the proportion of JIA patients having used SSZ was low (15%). Most ERA patients have peripheral joint involvement during the initial years and some of them develop axial disease later. Only a few patients present with axial symptoms and radiographic sacroiliitis within the first 2 to 3 years of disease [22]. Peripheral manifestations of the joint disease during the first years and better efficacy of MTX in uveitis could be reasons why MTX was preferred over SSZ as the first choice of treatment [23]. Another aspect in favour of methotrexate could be its easier once a week dosing. In ACR guidelines published after this study, TNFi use over MTX and SSZ is recommended in JIA and active enthesitis after treatment failure with NSAIDs [24]. However, in adults with peripheral spondyloarthritis conventional DMARDs are recommended with SSZ being the preferred option due to its demonstrated efficacy in this subgroup of patients [25]. In Finland, it would not be possible to prescribe TNFi before trying conventional DMARDs first due the reimbursement rules of the National Health Institute.

MTX and steroids were used more often by antigen positive patients, but still more than half of the patients had had biological medication at some point of the follow-up period, which supports the earlier findings suggesting the ineffectiveness of the traditional DMARDs in HLA-B27 positive patients [26].

Earlier studies have observed severity and chronicity of the HLA-B27 associated joint disease [20, 27–29]. In the present study, the HLA-B27 positivity correlated also with worse prognosis. Despite the new and more efficient therapies, still one third of antigen positive patients had active disease at 18-year follow-up visit, having either active joint disease, uveitis or both. Furthermore, 42% of antigen positive patients with active disease had ongoing biological medication.

One of the strengths of our prospective study with consecutive newly diagnosed JIA patients is that it has most likely included larger number of mild cases than referral-based studies, thus, minimizing the selection bias. However, one of the study’s limitations is the small sample size. Other limitations include the shortage of some variables and measurements that are used widely nowadays but were not available in the late 90s when the study was launched. In addition, in the first years of the study period biologics were sparse and the threshold to start them was far higher than today. In this study we also miss the data between 8 and 18 years, so we do not know how the disease course might have fluctuated during that period.

Regarding all the patients, both HLA-B27 positive and negative, it is important to notice that as high proportion of patients as one third was not in remission regardless, whether they were using DMARDs or not at 8-year follow-up. At 18 years, the percentage of patients with active disease was lower, but still 22% had an active disease. In 30% of these patients with active disease their biological medication had been discontinued at some point before the relapse and in 21% all their medications had been discontinued. This suggests that it is very important to carefully select the patients whose medication is discontinued. Similar findings on patients with active disease were reported in the study performed by Wallace et al. [30]. In addition, in a Norwegian 30-year follow-up study by Selvaag et al., 41% of the 176 JIA patients had active disease or were on medication after 30 years and 28% had a high symptom state [31]. Even in the era of biological medication, JIA patients still develop permanent joint damage and micrognatia, but it seems to be less frequent than in the past.

## Conclusions

The data of this study confirms the longstanding character of JIA, irrespective of the HLA-B27 antigen status. Therefore, high-quality ongoing care is beneficial, especially because the chronic activity of JIA may cause decreased quality of life, joint or ocular damage and need for surgery, implying that the disease duration is a strong predictor of an unfavorable disease outcome [4, 32]. The presence of HLA-B27 seems to be associated with a more severe and chronic disease course and need for more aggressive medical treatment. However, more information in larger cohorts on other potential prognostic markers is required to ensure the optimal care and treatment of JIA patients regardless of their age.

## Data Availability

The datasets used and/or analysed during the current study are available from the corresponding author on reasonable request.

## Declarations

Written informed consent was obtained from the parents of patients younger than 15 years and from the patients who were 15 years or older.

## Abbreviations

ANA: Antinuclear antibodies
AS: Ankylosing spondylitis
CBCT: Cone beam computed tomography
CRP: C-reactive protein
DMARDs: Disease-modifying antirheumatic drugs
ERA: Enthesitis-related arthritis
ESR: Erythrocyte sedimentation rate
HCQ: Hydroxychloroquine
IBD: Inflammatory bowel disease
JIA: Juvenile idiopathic arthritis
JPsA: Juvenile psoriatic arthritis
JSpA: Juvenile spondyloarthritis
MRI: Magnetic resonance imagining
MTX: Methotrexate
NSAIDs: Non-steroidal anti-inflammatory drugs
PsA: Psoriatic arthritis
ReA: Reactive arthritis
RF: Rheumatoid factor
SI joints: Sacroiliac joints
SpA: Spondyloarthritis
SSZ: Sulphasalazine
TMJ: Temporomandibular joint
TNFi: Tumor necrosis factor alpha inhibitor
